# Genome sequences of the 15 bluetongue virus vaccine strains incorporated in the South African live-attenuated vaccine

**DOI:** 10.1128/mra.00223-24

**Published:** 2024-05-21

**Authors:** Tendai A. M. Mlingo, Natasha Beeton-Kempen, M. Bethuel Nthangeni, Jacques Theron, Nobalanda B. Mokoena

**Affiliations:** 1 Department of Biochemistry, Genetics, and Microbiology, Faculty of Natural and Agricultural Sciences, University of Pretoria, Pretoria, South Africa; 2 Research and Development-Virology, Onderstepoort Biological Products, Pretoria, South Africa; 3 The Animal Health Group, TokaBio (PTY) LTD, Johannesburg, South Africa; Queens College, Queens, New York, USA

**Keywords:** vaccines, live-attenuated, Bluetongue virus

## Abstract

Bluetongue disease in endemic areas is predominantly controlled through vaccination with live-attenuated vaccines. Sequencing of the original master seed viruses used in the production of Onderstepoort Biological Products vaccine was conducted. Nucleotide identities of 82.97%–100% were obtained for all sequences when compared to South African reference strains.

## ANNOUNCEMENT

Bluetongue virus (BTV) is the prototype member of the *Orbivirus* genus (*Sedoreoviridae* family) (1) that causes disease in ruminants. The genome comprises 10 linear double-stranded RNA segments encapsidated by three protein layers (2). Bluetongue disease is controlled through vaccination with licensed inactivated and live-attenuated vaccines (LAV) (3). The LAV supplied by Onderstepoort Biological Products (OBP) has been used for decades in South Africa and comprises 15 serotypes (4) equally distributed in three bottles. Sequencing of plaque-purified vaccine strains has been reported (5, 6). However, legislation for veterinary biological registration stipulates a maximum of five passages from the master seed antigen during vaccine production (7). We describe the genome sequences of master seed antigens used in the OBP LAV production.

Virus strains were propagated on baby hamster kidney cells, and total RNA was extracted using Tri Reagent LS (Sigma). The Ion Total RNA-Seq Kit v2 (ThermoFisher Scientific) was applied to the viral RNA to fragment, hybridize, and ligate barcoded Ion Adapters. cDNA was synthesized from ligated RNA fragments and purified. The Ion PGM Template OT2 200 kit was used to generate template-positive Ion Sphere Particles containing enriched cDNA library with the Ion OneTouch 2 System. Sequencing was completed using the Ion PGM System at TokaBio (Pty) Ltd. The Illumina sequencing technology [Inqaba Biotechnical Industries (Pty) Ltd] was applied as an additional platform. The cDNA was synthesized using random hexamers or the full-length amplification of the cDNA method (8). Paired-end libraries were prepared by enzymatic fragmentation using the NEBNext Ultra II FS DNA Library Prep Kit. The cDNA ends were repaired, and specific adapter sequences and indexes were ligated to all fragments. Size selection was completed using AMPure XP Beads (Beckman Coulter) and quality controlled using the Agilent 2100 Bioanalyzer. Sequencing was performed on the NextSeq 500 instrument using the NextSeq 500/550 Mid-Output Kit v2.5 (2 × 150 bp) or the MiSeq instrument using the MiSeq Reagent kit v3 (2 × 300 bp). Quality control was performed using FastQC, and the Trimmomatic tool was applied for trimming adapters and low-quality reads.

Genome assembly was carried out on CLC Genomics Workbench 9.5.3 by mapping reads to reference strains. The full-length or coding-complete genome sizes and GC content are summarized in Table 1. The terminal ends were determined from data generated from both sequencing platforms. Mapped reads ranged from 1,544 to 8,344,163 across all serotypes using Illumina sequencing. The average read depth ranged from 7 to 40,359 with mean read lengths of ≤148 bases. On the Ion Torrent platform, mapped reads ranged from 3,655 to 55,002. Mean read lengths of ≤88 bases gave average read depths between 5 and 223. A pairwise alignment of the open reading frames of the 15 vaccine sequences with South African reference genomes (9–11) was carried out using BioEdit 7.2.5 (12) to determine nucleotide identities. Default parameters were used for all software unless otherwise specified. The nucleotide identities of bottle A serotypes were ≥82.97% for all segments. Bottle B and C serotypes had nucleotide identities of ≥99.49% and ≥92%, respectively.

**TABLE 1:** Accession numbers of the virus strains in the OBP BTV LAV

Type	Genome size bp	GC%	Seg-1	Seg-2	Seg-3	Seg-4	Seg-5	Seg-6	Seg-7	Seg-8	Seg-9	Seg-10	SRA accession number
1^*a*^	19,196	43.89	MZ130555	MZ130556	MZ130557	MZ130558	MZ130559	MZ130560	MZ130561	MZ130562	MZ130563	MZ130564	SRR21102004 ^*e*^
2^*c*^	19,162	43.75	MZ215851	MZ215852	MZ215853	MZ215854	MZ215855	MZ215856	MZ215857	MZ215858	MZ215859	MZ215860	SRR21859237 ^*f*^
3^*b*^	19,180	43.95	MZ395172	MZ395173	MZ395174	MZ395175	MZ395176	MZ395177	MZ395178	MZ395179	MZ395180	MZ395181	SRR27951317 ^*d*^ SRR27951318f
4^*a*^	19,188	44.07	MZ130565	MZ130566	MZ130567	MZ130568	MZ130569	MZ130570	MZ130571	MZ130572	MZ130573	MZ130574	SRR21390335 ^*e*^
5^*c*^	19,178	43.94	MZ215861	MZ215862	MZ215863	MZ215864	MZ215865	MZ215866	MZ215867	MZ215868	MZ215869	MZ215870	SRR21397265 ^*e*^
6^*a*^	19,180	43.94	MZ130575	MZ130576	MZ130577	MZ130578	MZ130579	MZ130580	MZ130581	MZ130582	MZ130583	MZ130584	SRR21391070 ^*e*^
7^*c*^	19,191	44.13	MZ215871	MZ215872	MZ215873	MZ215874	MZ215875	MZ215876	MZ215877	MZ215878	MZ215879	MZ215880	SRR21397283 ^*e*^
8^*b*^	19,186	44.06	MZ395182	MZ395183	MZ395184	MZ395185	MZ395186	MZ395187	MZ395188	MZ395189	MZ395190	MZ395191	SRR27915352 ^*d*^ SRR27926864 ^*f*^
9^*b*^	19,147	44.06	MZ395192	MZ395193	MZ395194	MZ395195	MZ395196	MZ395197	MZ395198	MZ395199	MZ395200	MZ395201	SRR27965401 ^*d*^ SRR27965562 ^*f*^
10^*b*^	19,157	43.87	MZ395202	MZ395203	MZ395204	MZ395205	MZ395206	MZ395207	MZ395208	MZ395209	MZ395210	MZ395211	SRR27919851 ^*d*^ SRR27944149 ^*f*^
11^*b*^	19,121	44.21	MZ395212	MZ395213	MZ395214	MZ395215	MZ395216	MZ395217	MZ395218	MZ395219	MZ395220	MZ395221	SRR27919922 ^*d*^ SRR27943927 ^*f*^
12^*a*^	19,170	43.90	MZ130585	MZ130586	MZ130587	MZ130588	MZ130589	MZ130590	MZ130591	MZ130592	MZ130593	MZ130594	SRR21391091 ^*e*^
13^*c*^	18,182	43.98	MZ215881	MZ215882	MZ215883	MZ215884	MZ215885	MZ215886	MZ215887	MZ215888	MZ215889	MZ215890	SRR27920106 ^*d*^ SRR27944117f
14^*a*^	19,180	44.05	MZ130595	MZ130596	MZ130597	MZ130598	MZ130599	MZ130600	MZ130601	MZ130602	MZ130603	MZ130604	SRR21397276 ^*e*^
19^*c*^	19,189	44.16	MZ215891	MZ215892	MZ215893	MZ215894	MZ215895	MZ215896	MZ215897	MZ215898	MZ215899	MZ215900	SRR27920286 ^*d*^ SRR27944147f

^*a*^ Bottle A.

^*b*^ Bottle B.

^*c*^ Bottle C.

^*d*^ Illumina NextSeq platform.

^*e*^ Illumina MiSeq.

^*f*^ Ion Torrent platform.

## Data Availability

The raw sequence data as well as assembled sequence data of the 15 serotypes were deposited in GenBank under BioProject number PRJNA718723. The accession numbers are summarised in Table 1. Detailed information on the sequencing data generated for each serotype such as nucleotide identity and the South African reference strain applied is available in Table S1 in figshare (https://doi.org/10.6084/m9.figshare.25611486).
